# Anxiety, Distress, and Depression in Elderly Rheumatoid Arthritis Patients

**DOI:** 10.31138/mjr.33.4.394

**Published:** 2022-12-31

**Authors:** Dimitrios Karokis, Dimitrios Karamanis, Sofia Xesfingi, Ioannis Antonopoulos, Eydokia Politi, Andreas Bounas, Chrysa Lykoura, Paraskevi Voulgari

**Affiliations:** 1 Rheumatologist, Private Practice, Patras, Greece,; 2 Adjunct Lecturer, School of Social Sciences, Hellenic Open University, Patras, Greece,; 3 Rheumatologist, Private Practice, Aigion, Greece,; 4 Rheumatologist, Private Practice, Arta, Greece,; 5 Rheumatologist, Private Practice, Patras, Greece,; 6 Trainee in Rheumatology, University Hospital Patras, Greece,; 7 Professor of Rheumatology, Department of Rheumatology, School of Health, Sciences, Faculty of Medicine, University of Ioannina, Greece

**Keywords:** rheumatoid arthritis, anxiety, depression, STAI, GHQ28, HAQ-DI, elderly

## Abstract

Rheumatoid arthritis (RA) and affective disorders (anxiety/depression) constitute important pathologies in the elderly population, and their coexistence creates synergistically increased problems in functional ability and quality of life of the patients.

**Purpose:**

Evaluation of anxiety, distress, and depression in elderly (≥65 years old) patients with RA.

**Patients – methods:**

114 patients from the cities of Patras, Arta and Ioannina (all located in Western Greece) were included. Demographics and medical information regarding RA were recorded, including disease duration, medication, previous treatments, disease activity measures, comorbidities etc. Patients answered the State-Trait Anxiety Inventory (STAI), General Health Questionnaire–28 (GHQ28) and Health Assessment Questionnaire -Disability Index (HAQ-DI) questionnaires, for evaluation of anxiety, general health and functional ability, respectively. Statistical analysis was made by using STATA.

**Results:**

88 women (78.07%) and 25 men (21.93%) with median age 70 years and median disease duration 10 years were studied. Female patients, with longer disease duration and higher disease activity, had statistically significant higher levels of anxiety, worse general health and decreased functional ability. A strongly significant association was found between the levels of anxiety and distress, with disease activity and functional inability.

**Conclusions:**

Levels of anxiety and distress are strongly associated with disease activity and functional inability in elderly patients with RA. Women with longer disease have higher levels of anxiety and distress. Controlling disease activity is of upmost importance for improvement of anxiety and distress and functional ability. Larger studies are needed for evaluation of anxiety and distress in elderly patients with RA.

## INTRODUCTION

Demographic aging and extension of life expectancy have increased the number of elderly people (≥65 years) worldwide, and their number is expected to reach about 88.5 million in 2050, comparing to 40.2 million in 2010.^1^ More than 20% of the elderly population suffers from some kind of psychiatric disorder,^2^ more often anxiety and depression, whereas the prevalence of depression in the elderly ranges from 14.4% to 48.5%.^3^ Rheumatoid arthritis (RA) is a chronic autoimmune inflammatory systemic disease, which can lead to significant structural damage and diminished quality of life.^4^ RA has a worldwide prevalence of 0.5–1%, which is obviously smaller in the southern countries comparing to the northern ones, and in rural areas comparing to urban.^5^ Epidemiological studies of RA in Greece have shown a prevalence of 0.57–0.67%.^6,7^ RA is 2–3 times more frequent in women. A strong genetic predisposition (“shared epitope”), as well as female sex, smoking, periodontal disease, obesity and chronic inhalation of small particles (dust, silica, asbestos etc) have been identified as risk factors for RA.^4^

Adverse prognostic factors for worse outcome of RA have been shown to be female sex, positive rheumatoid factor (RF) or/and anti-citrullinated protein antibodies (ACPA or anti-CCP), HLA DR-4 shared epitope, early erosions, increased indices of inflammation (Erythrocyte Sedimentation Rate, ESR and C-Reactive Protein (CRP), polyarthritic involvement, deranged functional ability, smoking, obesity, low socioeconomic status and comorbidities.^8^

RA patients can have several comorbidities. The most serious one is cardiovascular disease, which is the main cause for increased morbidity in RA patients and reduced life expectancy by 6–7 years.^9^ Other important comorbidities include infections, cancers, osteoporosis, mental and psychiatric disorders, whereas several side effects can be caused by the various therapeutic agents used for RA treatment.

A major effect of the existence of various comorbidities in patients with RA is polypharmacy. Both numbers of comorbidities and medications are increased with age and disease duration, with most of the RA patients receiving a considerable number of medications, apart from analgesics or other Over-the-counter (OTCs).^10^

More than one third of RA patients are over 65 years of age,^11^ but despite this fact, elderly patients are under-represented in clinical trials, mainly due to exclusion criteria.^12^ RA in the elderly is characterized by more acute onset, more systematic symptoms, more frequent involvement of big and proximal joints, and worse outcome. There are also a lot of comorbidities, polypharmacy, increased prevalence of depression and cognitive disturbances, increased cardiovascular risk, and increased risk for infections and cancers due to immunosenescense. There is also an increased risk of side effects from the various therapeutic agents, with a need for dose adjustment or drug withdrawal. In general, treating an elderly patient with RA sets a serious challenge and demands increased alertness.^11^

Several studies and meta-analyses have shown a 17–38% prevalence of depression in RA patients,^13^ with a relative risk of 1.46 comparing to general population.^14^ Anxiety is also frequent in patients with RA, with a prevalence of 16%, which is higher in patients with RA who have comorbid major depression than in either the general population of patients with RA or age-matched healthy controls.^15^ Anxiety and depression can cause a vicious circle of non-compliance, increased symptoms and exacerbations of the disease, deterioration of functional ability, more depression and anxiety etc.^16^ Depression is also responsible for an increased suicidal ideation in RA patients.^17^ It is thus obvious that co-existence of RA and anxiety/depression can cause synergistically augmented problems and further negatively affect quality of life in RA patients. The interaction of these disease entities in the elderly age group has not been adequately studied so far.

Our objective with this analysis was to estimate the levels of anxiety and perceived general health in elderly patients with RA, their relation with various demographic characteristics and medical data, and compare with relevant data from the literature.

## MATERIALS AND METHODS

### Patients-methods

A sample of 114 patients were included in the study, aged ≥ 65 years, during the period from 1/3/21 to 31/5/21. The patients came from the areas of Patras, Aigio, Ioannina, and Arta (all in Western Greece) and were recruited in private rheumatology practices, the outpatient rheumatology departments of the University Hospital of Ioannina and the University Hospital of Patras, and via patient associations. The patients were consecutively included, provided that they fulfilled the inclusion criteria (diagnosis of RA, age ≥ 65), and signed informed consent. They were asked to fill in the State-Trait Anxiety Inventory(STAI), General Health Questionnaire–28 (GHQ28) and Health Assessment Questionnaire (HAQ) questionnaires, whereas demographics and medical details (duration of disease, smoking, body mass index, current and past treatments for RA and other comorbidities, ESR, CRP, Clinical Disease Activity Index-CDAI) were completed by the main researcher or the other doctors.

### Questionnaires – measurement tools

For the estimation of anxiety, the Greek version of STAI was used. STAI is a general tool for measurement of anxiety, consisting of 20 questions about anxiety at present (STAI-S) and another 20 questions about anxiety as a characteristic of one’s personality (STAI-T). The answers are graded from 1 to 4, with the highest grade meaning more anxiety, and total and subcategories scores are created. The STAI was translated and weighted for the Greek population by Fountoulakis et al.^18^ Mental health was evaluated using the GHQ28 questionnaire, created by Goldberg and translated-weighted for the Greek population by Garyfallos et al.^19^

The GHQ28 estimates the existence and severity of non-psychotic mental symptoms. It consists of four groups of questions (seven questions in each group, scored 0–3) referring to somatic symptoms (A), anxiety and somnolence (B), everyday functional ability (C) and symptoms of depression (D). The highest possible score is 84, whereas a score above 24 is considered as a threshold to suspect non-psychotic mental symptoms and is characterized as “bad health-distress”.^20^

The HAQ-DI was used to assess the patients’ functional ability. Since it was originally developed in 1978 at Stanford University, has been widely used in RA (among several other chronic rheumatic and non-rheumatic diseases) to assess disability, whereas a higher HAQ score at the early stages of RA has been recognized as an adverse prognostic risk factor for more severe disease and worse outcome.^21^ The HAQ-DI questionnaire consists of 20 questions in 8 groups, referring to everyday living activities and scored by 0–3. The total score is divided by 8 and the higher scores correspond to heavier disability.^22^ Activity of the disease was assessed using the ESR and CRP values, as well as the CDAI score.

### Outcomes and statistical analysis

Patients were classified per gender, HAQ-DI levels (Low category refers to values between 0 and 1, while High category to values between 1.1 and 3) and CDAI levels (Low category refers to values between 0 and 10, while High category to values between 10.1 and 76). The primary outcomes were the levels of reported STAI (anxiety) and GHQ28 (general health / distress). Secondary ones included the levels of the respective sub-indexes, that is STAI-T, STAI-S, somatic symptoms (GHQ28-A), anxiety and somnolence (GHQ28-B), everyday functional ability (GHQ28-C), and symptoms of depression (GHQ28-D). In all cases and for consistency, high levels of the reported outcomes were considered index values above median (50th percentile) (see **Table 3**). Continuous variables are presented as median with interquartile range (IQR) and categorical data as absolute with relative frequencies. The normality of the data was evaluated using the Skewness/Kurtosis test. The Student’s t-test (for normally distributed variables) or Mann-Whitney test (for non-normally distributed variables) was used to compare the continuous variables, while chi-square test or Fisher’s exact test (when the expected values within a cell was below five) for discrete variables. We applied univariate logistic regression analyses and one multivariate model for each dichotomous outcome, that included baseline characteristics that were found significant (p < 0.05) in the univariate. Logistic regressions’ results are reported as odds ratios (OR) with the 95% confidence interval (CI). A nominal *p*-value < 0.05 was considered statistically significant. Internal reliability of the questionnaires was assessed and verified by Cronbach’s alpha. The analysis was performed using STATA software (version 14.1).

**Table 3.** STAI and GHQ28 Outcomes.

**Variables**	**All patients**	**Gender**			**HAQ-DI**			**CDAI**		
			
		**Man**	**Woman**	***p*-value**	**Low**	**High**	***p*-value**	**Low**	**High**	***p*-value**
	N=114	N=25	N=89		N=79	N=35		N=70	N=44	
**STAI**										
median (IQR)	87.0 (67.0–100.0)	79.0 (58.0–89.0)	89.0 (71.0–104.0)	**0.012**	84.0 (66.0–98.0)	93.0 (73.0–110.0)	0.065	80.0 (63.0–95.0)	94.0 (78.5–111.0)	**0.005**
High levels (>87) - n(%)	54 (47.37)	8 (32.00)	46 (51.69)	0.082	34 (43.04)	20 (57.14)	0.164	26 (37.14)	28 (63.64)	**0.006**
Very High levels (>100) - n(%)	28 (24.56)	2 (8.00)	26 (29.21)	**0.029**	15 (18.99)	13 (37.14)	**0.038**	14 (20.00)	14 (31.82)	0.154
**STAI-S**										
median (IQR)	43.0 (33.0–51.0)	38.0 (31.0–47.0)	45.0 (35.0–55.0)	**0.020**	43.0 (33.0–51.0)	47.0 (36.0–57.0)	0.208	41.0 (32.0–50.0)	48.5 (36.5–57.5)	**0.025**
High levels (>43) - n(%)	56 (49.12)	10 (40.00)	46 (51.69)	0.302	38 (48.10)	18 (51.43)	0.743	28 (40.00)	28 (63.64)	**0.014**
Very High levels (>51) - n(%)	28 (24.56)	2 (8.00)	26 (29.21)	**0.029**	16 (20.25)	12 (34.29)	0.108	13 (18.57)	15 (34.09)	**0.019**
**STAI-T**										
median (IQR)	42.0 (34.0–50.0)	37.0 (31.0–42.0)	43.0 (36.0–51.0)	**0.018**	40.0 (32.0–48.0)	47.0 (36.0–55.0)	**0.028**	39.0 (31.0–47.0)	47.0 (39.0–53.5)	**0.003**
High levels (>42) - n(%)	53 (46.49)	6 (24.00)	47 (52.81)	**0.011**	31 (39.24)	22 (62.86)	**0.020**	26 (37.14)	27 (61.36)	**0.012**
Very High levels (>50) - n(%)	27 (23.68)	4 (16.00)	23 (25.84)	0.306	15 (18.99)	12 (34.29)	0.076	11 (15.71)	16 (36.36)	**0.012**
**GHQ28**										
median (IQR)	22.0 (16.0–31.0)	18.0 (11.0–22.0)	23.0 (17.0–32.0)	**0.007**	19.0 (15.0–25.0)	31.0 (22.0–41.0)	**<0.001**	19.0 (15.0–25.0)	27.0 (19.0–33.5)	**0.007**
High levels (>22) - n(%)	54 (47.37)	6 (24.00)	48 (53.93)	**0.008**	29 (36.71)	25 (71.43)	**0.001**	25 (35.71)	29 (65.91)	**0.002**
Very High levels (31) - n(%)	25 (21.93)	2 (8.00)	23 (25.84)	0.057	11 (13.92)	14 (40.00)	**0.002**	13 (18.57)	12 (27.27)	0.274
**GHQ28-A**/**Somatic Symptoms**										
median (IQR)	6.0 (4.0–9.0)	5.0 (3.0–6.0)	6.0 (5.0–10.0)	**0.013**	5.0 (3.0–7.0)	9.0 (7.0–12.0)	**<0.001**	5.0 (3.0–7.0)	7.0 (5.0–11.0)	**0.005**
High levels (>6) - n(%)	50 (43.86)	6 (24.00)	44 (49.44)	**0.024**	22 (27.85)	28 (80.00)	**<0.001**	22 (31.43)	28 (63.64)	**0.001**
Very High levels (>9) - n(%)	26 (22.81)	2 (8.00)	24 (26.97)	**0.046**	10 (12.66)	16 (45.71)	**<0.001**	12 (17.14)	14 (31.82)	0.069
**GHQ28-B / Anxiety/Insomnia**										
median (IQR)	6.0 (4.0–10.0)	4.0 (3.0–7.0)	7.0 (4.0–10.0)	**0.038**	6.0 (3.0–8.0)	8.0 (6.0–13.0)	**0.001**	6.0 (3.0–8.0)	7.0 (4.5–10.5)	**0.033**
High levels (>6) - n(%)	53 (46.49)	7 (28.00)	46 (51.69)	**0.036**	31 (39.24)	22 (62.86)	**0.020**	26 (37.14)	27 (61.36)	**0.012**
Very High levels (>10) - n(%)	22 (19.30)	2 (8.00)	20 (22.47)	0.105	10 (12.66)	12 (34.29)	**0.007**	11 (15.71)	11 (25.00)	0.221
**GHQ28-C / Social Dysfunction**										
median (IQR)	8.0 (6.0–11.0)	7.0 (5.0–9.0)	8.0 (7.0–11.0)	0.140	7.0 (6.0–10.0)	10.0 (8.0–14.0)	**<0.001**	7.0 (6.0–9.0)	9.5 (7.0–13.0)	**0.003**
High levels (>8) - n(%)	50 (43.86)	8 (32.00)	42 (47.19)	0.176	27 (34.18)	23 (65.71)	**0.002**	23 (32.86)	27 (61.36)	**0.003**
Very High levels (>11) - n(%)	23 (20.18)	5 (20.00)	18 (20.22)	0.980	11 (13.92)	12 (34.29)	**0.012**	9 (12.86)	14 (31.82)	**0.014**
**GHQ28-D / Severe Depression**										
median (IQR)	1.0 (0.0–3.0)	1.0 (0.0–1.0)	1.0 (0.0–3.0)	0.088	1.0 (0.0–2.0)	1.0 (0.0–5.0)	0.087	1.0 (0.0–2.0)	1.0 (0.0–3.0)	0.818
High levels (>1) - n(%)	42 (36.84)	5 (20.00)	37 (41.57)	**0.048**	27 (34.18)	15 (42.86)	0.376	23 (32.86)	19 (43.18)	0.266
Very High levels (>3) - n(%)	21 (18.42)	3 (12.00)	18 (20.22)	0.349	12 (15.19)	9 (25.71)	0.181	13 (18.57)	8 (18.18)	0.958

*p*-values refer to T-test/Mann-Whitney test or Chi-square test/Fisher’s exact test. High levels for each outcome were considered values above 50% percentile (median value). Very high levels were considered values above 75% percentile. HAQ-DI Low category refers to values between 0 and 1, while High category to values between 1.1 and 3. CDAI Low category refers to values between 0 and 10, while High category to values between 10.1 and 76. IQR: interquartile range; n: number.

## RESULTS

Patients’ demographics and medical/clinical characteristics are shown in **Tables 1** and **2**. Eighty nine (89) patients (78.07%) were females and twenty-five (25) patients (21.93 %) males. Median age was 70 years (IQR 66 - 74). Median disease duration was 10 years (females 11 years, males 5 years). Median BMI was 27.3 (IQR 25.0 – 30.4) and 49 patients (42.98%) were RF positive with significant differences between genders [4 (24%) men vs 43 (48.31%) women *p* = 0.030], and 37 patients (32.74%) were CCP positive. Median CRP was 0.4 (IQR 0.2 - 0.8), while median HAQ-DI was 0.0 (IQR 0.0 - 0.8) for men vs. 0.9 (IQR 0.3 - 1.4) for women (*p* = 0.001). Median CDAI was 8.5 (IQR 6 - 13) and above half of the patients belonged in the Low CDAI category (50.88%).

**Table 1.** Demographic characteristics.

**Variables**	**All patients**	**Gender**			**HAQ-DI**			**CDAI**		
			
		**Man**	**Woman**	***p*-value**	**Low**	**High**	***p*-value**	**Low**	**High**	***p*-value**
	N=114	N=25	N=89		N=79	N=35		N=70	N=44	
**Demographics**										
Gender - n(%)				**<0.001**			**0.001**			0.090
Male	25 (21.93)	25 (100.00)	0 (0.00)		24 (30.38)	1 (2.86)		19 (27.14)	6 (13.64)	
Female	89 (78.07)	0 (0.00)	89 (100.00)		55 (69.62)	34 (97.14)		51 (72.86)	38 (86.36)	
Age in years - median (IQR)	70.0 (66.0–74.0)	70.0 (66.0–73.0)	70.0 (65.0–75.0)	0.498	70.0 (66.0–74.0)	70.0 (65.0–79.0)	0.071	69.0 (66.0–74.0)	70.0 (66.0–76.5)	0.370
Marital Status - n(%)				**0.024**			**0.003**			0.521
Divorced/Single/Widow	45 (39.47)	5 (20.00)	40 (44.94)		24 (30.38)	21 (60.00)		26 (37.14)	19 (43.18)	
Married	69 (60.53)	20 (80.00)	49 (55.06)		55 (69.62)	14 (40.00)		44 (62.86)	25 (56.82)	
Roommates - n(%)				0.749			0.899			0.463
0	34 (29.82)	6 (24.00)	28 (31.46)		23 (29.11)	11 (31.43)		18 (25.71)	16 (36.36)	
1	36 (31.58)	9 (36.00)	27 (30.34)		26 (32.91)	10 (28.57)		24 (34.29)	12 (27.27)	
2–4	44 (38.60)	10 (40.00)	34 (38.20)		30 (37.97)	14 (40.00)		28 (40.00)	16 (36.36)	
Education (ISCED Levels) - n(%)				0.718			0.919			0.435
0–1 - Primary	46 (40.35)	11 (44.00)	35 (39.33)		31 (39.24)	15 (42.86)		26 (37.14)	20 (45.45)	
2–4 - Secondary	43 (37.72)	10 (40.00)	33 (37.08)		30 (37.97)	13 (37.14)		26 (37.14)	17 (38.64)	
5–8 - Tertiary	25 (21.93)	4 (16.00)	21 (23.60)		18 (22.78)	7 (20.00)		18 (25.71)	7 (15.91)	
Income - n(%)				0.117			0.978			**0.035**
Low (≤550€)	33 (28.95)	4 (16.00)	29 (32.58)		23 (29.11)	10 (28.57)		21 (30.00)	12 (27.27)	
Medium (551–1000€)	44 (38.60)	9 (36.00)	35 (39.33)		30 (37.97)	14 (40.00)		21 (30.00)	23 (52.27)	
High (≥1001€)	37 (32.46)	12 (48.00)	25 (28.09)		26 (32.91)	11 (31.43)		28 (40.00)	9 (20.45)	
Residency - n(%)				0.379			0.064			0.113
Urban	81 (71.05)	16 (64.00)	65 (73.03)		52 (65.82)	29 (82.86)		46 (65.71)	35 (79.55)	
Rural	33 (28.95)	9 (36.00)	24 (26.97)		27 (34.18)	6 (17.14)		24 (34.29)	9 (20.45)	

*p*-values refer to T-test/Mann-Whitney test or Chi-square test/Fisher’s exact test. HAQ-DI Low category refers to values between 0 and 1, while High category to values between 1.1 and 3. CDAI Low category refers to values between 0 and 10, while High category to values between 10.1 and 76.

**Table 2.** Medical and clinical characteristics.

**Variables**	**All patients**	**Gender**			**HAQ-DI**			**CDAI**		
			
		**Man**	**Woman**	***p*-value**	**Low**	**High**	***p*-value**	**Low**	**High**	***p*-value**
	N=114	N=25	N=89		N=79	N=35		N=70	N=44	
BMI in kg/m^2^- median (IQR)	27.3 (25.0–30.4)	26.9 (25.5–31.1)	28.1 (25.0–30.4)	0.405	26.4 (24.7–30.0)	29.3 (26.0–31.2)	0.103	27.4 (24.8–30.4)	26.8 (25.2–30.6)	0.589
BMI Category - n(%)				0.954			0.162			0.548
<25 kg/m^2^	27 (23.68)	6 (24.00)	21 (23.60)		22 (27.85)	5 (14.29)		19 (27.14)	8 (18.18)	
25–29.9 kg/m^2^	53 (46.49)	11 (44.00)	42 (47.19)		37 (46.84)	16 (45.71)		31 (44.29)	22 (50.00)	
≥30 kg/m^2^	34 (29.82)	8 (32.00)	26 (29.21)		20 (25.32)	14 (40.00)		20 (28.57)	14 (31.82)	
Smoker - n(%)	24 (21.05)	4 (16.00)	20 (22.47)	0.483	18 (22.78)	6 (17.14)	0.496	15 (21.43)	9 (20.45)	0.901
Previous Smoker - n(%)	20 (17.54)	8 (32.00)	12 (13.48)	**0.031**	17 (21.52)	3 (8.57)	0.094	15 (21.43)	5 (11.36)	0.169
Years of disease - median (IQR)	10.0 (5.0–15.0)	5.0 (3.0–10.0)	11.0 (5.0–17.0)	**0.002**	10.0 (3.0–15.0)	13.0 (8.0–21.0)	**0.035**	10.0 (4.0–15.0)	11.5 (6.0–15.0)	0.182
Hypertension - n(%)	64 (56.14)	16 (64.00)	48 (53.93)	0.370	47 (59.49)	17 (48.57)	0.278	37 (52.86)	27 (61.36)	0.373
Diabetes - n(%)	20 (17.54)	6 (24.00)	14 (15.73)	0.337	12 (15.19)	8 (22.86)	0.321	13 (18.57)	7 (15.91)	0.716
Thyroid disease - n(%)	25 (21.93)	3 (12.00)	22 (24.72)	0.174	18 (22.78)	7 (20.00)	0.740	11 (15.71)	14 (31.82)	**0.043**
Cancer - n(%)	4 (3.51)	0 (0.00)	4 (4.49)	0.281	2 (2.53)	2 (5.71)	0.394	1 (1.43)	3 (6.82)	0.128
Lipidaemia - n(%)	45 (39.47)	13 (52.00)	32 (35.96)	0.147	29 (36.71)	16 (45.71)	0.364	29 (41.43)	16 (36.36)	0.590
Osteoporosis - n(%)	27 (23.68)	2 (8.00)	25 (28.09)	0.037	18 (22.78)	9 (25.71)	0.734	17 (24.29)	10 (22.73)	0.849
Inflamm Bowel Disease - n(%)	3 (2.63)	0 (0.00)	3 (3.37)	0.352	2 (2.53)	1 (2.86)	0.920	0 (0.00)	3 (6.82)	**0.027**
Fibromyalgia - n(%)	18 (15.79)	1 (4.00)	17 (19.10)	0.067	9 (11.39)	9 (25.71)	0.053	6 (8.57)	12 (27.27)	**0.008**
COPD - n(%)	12 (10.53)	3 (12.00)	9 (10.11)	0.786	6 (7.59)	6 (17.14)	0.125	6 (8.57)	6 (13.64)	0.391
No. of Comorbidities - n(%)				0.835			0.219			**0.006**
0–1	32 (28.07)	8 (32.00)	24 (26.97)		26 (32.91)	6 (17.14)		26 (37.14)	6 (13.64)	
2–3	55 (48.25)	12 (48.00)	43 (48.31)		36 (45.57)	19 (54.29)		33 (47.14)	22 (50.00)	
4–7	27 (23.68)	5 (20.00)	22 (24.72)		17 (21.52)	10 (28.57)		11 (15.71)	16 (36.36)	
TKE - median (IQR)	19.5 (10.0–26.0)	16.0 (8.0–28.0)	20.0 (12.0–26.0)	0.507	18.0 (10.0–28.0)	20.0 (13.0–26.0)	0.948	18.5 (10.0–25.0)	20.5 (13.0–31.0)	**0.010**
CRP - median (IQR)	0.4 (0.2–0.8)	0.3 (0.2–0.5)	0.4 (0.2–0.8)	0.885	0.4 (0.2–0.7)	0.5 (0.2–0.9)	0.695	0.4 (0.2–0.7)	0.5 (0.2–1.0)	**0.005**
RF - n(%)	49 (42.98)	6 (24.00)	43 (48.31)	**0.030**	35 (44.30)	14 (40.00)	0.671	30 (42.86)	19 (43.18)	0.973
CCP - n(%)	38 (33.33)	5 (20.00)	33 (37.08)	0.111	27 (34.18)	11 (31.43)	0.776	22 (31.43)	16 (36.36)	0.590
HAQ-DI - median (IQR)	0.8 (0.1–1.3)	0.0 (0.0–0.8)	0.9 (0.3–1.4)	**0.001**	0.5 (0.0–0.9)	1.5 (1.3–2.0)	**<0.001**	0.8 (0.0–1.0)	1.0 (0.3–1.5)	0.107
HAQ-DI Categories - n(%)				**0.003**			**<0.001**			0.105
0–1	79 (69.30)	24 (96.00)	55 (61.80)		79 (100.00)	0 (0.00)		53 (75.71)	26 (59.09)	
1–2	29 (25.44)	0 (0.00)	29 (32.58)		0 (0.00)	29 (82.86)		13 (18.57)	16 (36.36)	
2–3	6 (5.26)	1 (4.00)	5 (5.62)		0 (0.00)	6 (17.14)		4 (5.71)	2 (4.55)	
CDAI - median (IQR)	8.5 (6.0–13.0)	8.0 (3.0–10.0)	10.0 (7.0–13.0)	0.063	8.0 (4.0–11.0)	11.0 (8.0–18.0)	**<0.001**	7.0 (3.5–8.0)	14.5 (11.5–20.3)	**<0.001**
CDAI Categories - n(%)				0.195			**0.033**			**<0.001**
Remission (0.0 – 2.8)	12 (10.53)	5 (20.00)	7 (7.87)		11 (13.92)	1 (2.86)		12 (17.14)	0 (0.00)	
Low (2.9 – 10.0)	58 (50.88)	14 (56.00)	44 (49.44)		42 (53.16)	16 (45.71)		58 (82.86)	0 (0.00)	
Moderate (10.1 – 22.0)	35 (30.70)	5 (20.00)	30 (33.71)		23 (29.11)	12 (34.29)		0 (0.00)	35 (79.55)	
High (22.1 – 76.0)	9 (7.89)	1 (4.00)	8 (8.99)		3 (3.80)	6 (17.14)		0 (0.00)	9 (20.45)	

*p*-values refer to T-test/Mann-Whitney test or Chi-square test/Fisher’s exact test. HAQ-DI Low category refers to values between 0 and 1, while High category to values between 1.1 and 3. CDAI Low category refers to values between 0 and 10, while High category to values between 10.1 and 76.

IQR: interquartile range; BMI: body mass index; kg: kilogram; m: meter; n: number; COPD: chronic obstructive pulmonary disease; ESR: Erythrocyte Sedimentation Rate; CRP: C-reactive protein; RF: Rheumatoid Factor; CCP: Cyclic Citrullinated Peptide; HAQ-DI: Health Assessment Questionnaire Disability Index; CDAI: Clinical Disease Activity Index.

Current and previous treatments for their disease (RA) are reported in **Supplementary Table S1**. Methotrexate (52.63%), glucocorticoids (43.86%), anti-TNF (22.81%) were the three most common current treatments. The median number of treatments was 2 (IQR 1 – 2), whereas the hydroxychloroquine was significant different between Low and High HAQ-DI Categories (16.46% vs. 34.29%, *p* = 0.034).

**Figure 1.**
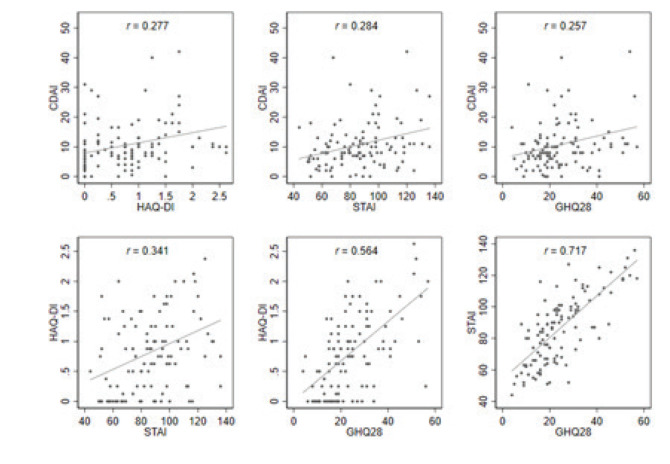
Scatter plots for HAQ-DI, CDAI, STAI and GHQ28.

Primary and secondary outcomes are reported in **Table 3**. The median values for the outcomes were respectively: STAI 87 (IQR 67 – 100), STAI-S 43 (IQR 33 – 51), STAI-T 42 (IQR 34 – 54), GHQ28 22 (IQR 16 – 31), GHQ28-A 6 (IQR 4 – 9), GHQ28-B 6 (IQR 4 – 10), GHQ28-C 8 (IQR 6 – 11), and GHQ28-D 1 (IQR 0 – 3). For each outcome/variable, two dichotomous versions, specifically high levels (values above median or 50th percentile) and very high levels (values above 75th percentile), are also presented. Results concerning the significant differences between genders, HAQ-DI levels and CDAI levels for all three versions of the outcomes are overall the same. Female patients had on average statistically significantly worse (higher) scores for all outcomes, except the Social Dysfunction (GHQ28-C). With respect to different levels of HAQ-DI, there is evidence for significant different values for all outcomes except STAI-S and Severe Depression (GHQ28-D), while different levels of CDAI are associated with all outcomes except Severe Depression (GHQ28-D). High positive correlations of HAQ-DI and CDAI with the primary outcomes are depicted in **Figure 1**.

The univariate associations with all outcomes were examined for all baseline demographic and clinical characteristics, and treatments (**Supplementary Table S2**). Female gender and longer duration of the disease were associated with worse reported levels for most of the outcomes. Interestingly, married patients reported significantly better social function (GHQ28-C). The following variables were not shown to be statistically significant in most of the univariate associations: Number of roommates, education level, income, residence, obesity and the comorbidities. ESR is found to be positively associated with all anxiety indexes (STAI, STAI-S, STAI-T), while higher number of treatments is found to have significant associations with high levels of anxiety (STAI), distress (GHQ28), somatic symptoms (GHQ28-A), and social disfunction (GHQ28-C). Higher functional disability (HAQ-DI) and disease activity (CDAI) were found to be strongly associated with worse reported outcomes and multivariate analysis (**Table 4**) verified this association in most of the cases.

**Table 4.** Multivariate logistic regression analyses.

**Models:**	**High STAI**	**High STAI-S**	**High STAI-T**	**High GHQ28**	**High GHQ28-A Somatic Symptoms**	**High GHQ28-B Anxiety/insomnia**	**High GHQ28-C Social Dysfunction**	**High GHQ28-D Severe depression**
	(1)	(2)	(3)	(4)	(5)	(6)	(7)	(8)
	OR, 95% CI, p-value	OR, 95% CI, p-value	OR, 95% CI, p-value	OR, 95% CI, p-value	OR, 95% CI, p-value	OR, 95% CI, p-value	OR, 95% CI, p-value	OR, 95% CI, p-value
**Submodel (a)**								
HAQ-DI	1.88 (0.96 - 3.67) p=0.064	1.68 (0.90 - 3.14) p=0.104	**2.92 (1.35 - 6.36) p=0.007**	**3.13 (1.43 - 6.85) p=0.004**	**9.70 (3.08 - 30.62) p<0.001**	**2.00 (1.07 - 3.76) p=0.031**	**2.72 (1.21 - 6.13) p=0.015**	1.80 (0.91 - 3.54) p=0.090
CDAI	1.02 (0.96 - 1.08) p=0.542	1.03 (0.98 - 1.09) p=0.227	1.00 (0.93 - 1.08) p=0.924	1.05 (0.99 - 1.11) p=0.120	**1.09 (1.02 - 1.17) p=0.012**	1.04 (0.98 - 1.09) p=0.201	**1.05 (1.00 - 1.11) p=0.042**	0.98 (0.92 - 1.04) p=0.485
**Submodel (b)**								
HAQ-DI scale	1.12 (0.46 - 2.74) p=0.796	0.90 (0.35 - 2.31) p=0.826	1.58 (0.59 - 4.21) p=0.364	2.32 (0.84 - 6.42) p=0.104	**9.58 (3.31 - 27.72) p<0.001**	1.97 (0.80 - 4.82) p=0.138	2.19 (0.75 - 6.40) p=0.152	1.15 (0.47 - 2.83) p=0.753
CDAI scale	2.13 (0.87 - 5.24) p=0.098	**2.65 (1.05 - 6.68) p=0.039**	1.79 (0.72 - 4.40) p=0.208	**3.03 (1.29 - 7.14) p=0.011**	**3.77 (1.44 - 9.87) p=0.007**	**2.30 (1.02 - 5.18) p=0.044**	**3.02 (1.12 - 8.17) p=0.029**	1.27 (0.56 - 2.88) p=0.562

**Model (1)** adjusted for previous smoking, years of disease, ESR, anti-TNF and number of treatments. **Model (2)** adjusted for age, number of roommates, years of disease, TKE, anti-TNF and anti-IL6. **Model (3)** adjusted for gender, previous smoking, COPD and TKE. **Model (4)** adjusted for gender, years of disease and number of treatments. **Model (5)** adjusted for gender, residency, osteoporosis, fibromyalgia and number of treatments. **Model (6)** adjusted for gender. **Model (7)** adjusted for age, marital status, income, anti-TNF and number of treatments. **Model (8)** adjusted for years of disease and anti-TNF. In **Submodel (a)** HAQ and CDAI are introduced to the models (1) to (8) as continuous variables. In **Submodel (b)** HAQ and CDAI are introduced to the models (1) to (8) as a two-scales categorical variable.

HAQ-DI: Health Assessment Questionnaire Disability Index; CDAI: Clinical Disease Activity Index.

## DISCUSSION

The patients in our study had some interesting characteristics. Female/male ratio was 3.5/1, similar to the usual and expected ratio. RF positivity was about 43% and ACPA positivity was about 33%, quite lower comparing to the literature.^8^ RA in the elderly population has been shown to have somewhat lower seropositivity,^11^ but not at such a degree. There is no obvious reason for this finding in the patients in our study, but it could be due to chance and/or the small number of participants. The patients had generally a long median disease duration (10 years) and quite low disease activity (evaluated by CDAI), which can be considered as a realistic situation given the long disease duration and explained as a result of a proper therapeutic strategy.

The patients in our study were taking a median of 2 medications for their disease, and most of them had 2–3 comorbidities (48.25%) with a median of 3 additional medications, consistent with data from the literature.^10^ Female patients in our study had significantly higher anxiety and distress, and worse functional ability. Obviously, older age and longer disease duration contributed to this difference. Additionally - and expectedly, adverse risk factors were age, higher disease activity, need for intense treatment (biologics, corticosteroids), and number of treatments.

These findings are quite consistent with data from the literature, where disease activity is a main factor of anxiety and depression, and the close relationship of anxiety/depression and reduced functional ability in a vicious circle of mutual deterioration, is also evident in the findings of our study.^14,16,23,24^ Older RA patients are known to experience more significant impairment of health-related quality of life compared to younger patients, and psychological distress is a considerable factor contributing to this.^25^ Additional finding of the present study is the association of anxiety and distress with gender and disease duration.

The increased prevalence of anxiety, distress, and depression in RA patients is largely reasonable and expected, as the RA patient has to manage the psychological load of a chronic disease, the derangement or loss of their personal and/or social roles, problems at work, compliance to treatment, medication side effects etc. Anxiety and depression have a negative effect on functional ability, adding on the inability caused by structural damage and chronic pain and fatigue.^14,26^ Proinflammatory cytokines (ie, Tumour necrosis factor [TNFa], Interleukin [IL]-1, IL-6), which circulate in abundance in patients with RA, can affect certain brain areas which relate to depression, neuroendocrine function and metabolism of neurotransmitters.^14^ Cases of acute RA evolution after a major stress, have indicated the possibility of common immunological mechanisms between the two entities, and a reverse pathophysiological relation, which is still being investigated.^24^

Limitation of this study is the relatively small number of patients, (due to the strict time-frame, as this study was conducted for a thesis in a Master’s programme in Management of Aging and Chronic Diseases, Hellenic Open University), which in turn might be responsible for particular characteristics of the patient population, ie, RF-ACPA positivity, etc.

## CONCLUSION

The strong relationship between anxiety, distress and depression with disease activity and functional (in) ability, was confirmed in this study. Females of older age and with longer disease duration had significantly higher levels of anxiety and distress. Control of disease activity is of upmost importance for reducing anxiety/distress symptoms and improving functional ability. This particular age group of RA patients (elderly ≥65 years) is very interesting and presents specific particularities. Larger and more robust studies to research on anxiety, depression, and mental health generally in this patient population would be extremely useful.

## Figures and Tables

**Supplementary Table S1.** Treatments and previous treatments.

**Variables**	**All patients**	**Gender**			**HAQ-DI**			**CDAI**		
			
		**Man**	**Woman**	***p*-value**	**Low**	**High**	***p*-value**	**Low**	**High**	***p*-value**
	N=114	N=25	N=89		N=79	N=35		N=70	N=44	
**Treatments - n(%)**										
Hydroxychloroquine	25 (21.93)	3 (12.00)	22 (24.72)	0.174	13 (16.46)	12 (34.29)	**0.034**	19 (27.14)	6 (13.64)	0.090
Methotrexate	60 (52.63)	18 (72.00)	42 (47.19)	**0.028**	44 (55.70)	16 (45.71)	0.325	40 (57.14)	20 (45.45)	0.224
Leflunomide	5 (4.39)	1 (4.00)	4 (4.49)	0.915	3 (3.80)	2 (5.71)	0.645	3 (4.29)	2 (4.55)	0.947
anti-TNF	26 (22.81)	4 (16.00)	22 (24.72)	0.359	17 (21.52)	9 (25.71)	0.622	12 (17.14)	14 (31.82)	0.069
anti-IL6	11 (9.65)	1 (4.00)	10 (11.24)	0.279	8 (10.13)	3 (8.57)	0.795	7 (10.00)	4 (9.09)	0.873
anti-CTLA4	1 (0.88)	0 (0.00)	1 (1.12)	0.594	1 (1.27)	0 (0.00)	0.504	0 (0.00)	1 (2.27)	0.205
Jak-inhibitors	5 (4.39)	3 (12.00)	2 (2.25)	0.069	4 (5.06)	1 (2.86)	0.596	2 (2.86)	3 (6.82)	0.315
anti-CD20	5 (4.42)	0 (0.00)	5 (5.68)	0.223	2 (2.53)	3 (8.82)	0.136	1 (1.45)	4 (9.09)	0.054
glucocorticoids	50 (43.86)	11 (44.00)	39 (43.82)	0.987	33 (41.77)	17 (48.57)	0.500	29 (41.43)	21 (47.73)	0.509
No. of Treatments - median (IQR)	2.0 (1.0–2.0)	2.0 (1.0–2.0)	2.0 (1.0–2.0)	0.912	2.0 (1.0–2.0)	2.0 (1.0–3.0)	0.124	2.0 (1.0–2.0)	2.0 (1.0–2.0)	0.583
**Previous Treatments - n(%)**										
Hydroxychloroquine	16 (14.04)	0 (0.00)	16 (17.98)	**0.022**	11 (13.92)	5 (14.29)	0.959	7 (10.00)	9 (20.45)	0.118
Methotrexate	46 (40.35)	6 (24.00)	40 (44.94)	0.059	29 (36.71)	17 (48.57)	0.234	24 (34.29)	22 (50.00)	0.096
Leflunomide	14 (12.28)	1 (4.00)	13 (14.61)	0.153	10 (12.66)	4 (11.43)	0.854	6 (8.57)	8 (18.18)	0.128
anti-TNF	22 (19.30)	3 (12.00)	19 (21.35)	0.295	10 (12.66)	12 (34.29)	**0.007**	11 (15.71)	11 (25.00)	0.221
anti-IL6	5 (4.39)	0 (0.00)	5 (5.62)	0.226	3 (3.80)	2 (5.71)	0.645	0 (0.00)	5 (11.36)	**0.007**
anti-CTLA4	5 (4.39)	0 (0.00)	5 (5.62)	0.226	3 (3.80)	2 (5.71)	0.645	2 (2.86)	3 (6.82)	0.315
Jak-inhibitors	1 (0.88)	0 (0.00)	1 (1.12)	0.594	0 (0.00)	1 (2.86)	0.131	0 (0.00)	1 (2.27)	0.205
anti-CD20	0 (0.00)	0 (0.00)	0 (0.00)	-	0 (0.00)	0 (0.00)	-	0 (0.00)	0 (0.00)	-
glucocorticoids	58 (50.88)	11 (44.00)	47 (52.81)	0.436	43 (54.43)	15 (42.86)	0.254	35 (50.00)	23 (52.27)	0.813
No. of Previous treatments - median (IQR)	1.0 (0.0–2.0)	1.0 (0.0–1.0)	2.0 (0.0–2.0)	**0.007**	1.0 (0.0–2.0)	2.0 (0.0–2.0)	0.253	1.0 (0.0–2.0)	2.0 (0.0–3.0)	**0.010**
No. of Medicines - median (IQR)	3.0 (2.0–4.0)	2.0 (2.0–5.0)	3.0 (2.0–4.0)	0.711	2.0 (2.0–5.0)	3.0 (2.0–4.0)	0.174	2.0 (2.0–4.0)	4.0 (2.5–6.0)	<0.001

*p*-values refer to T-test/Mann-Whitney test or Chi-square test/Fisher’s exact test. HAQ-DI Low category refers to values between 0 and 1, while High category to values between 1.1 and 3. CDAI Low category refers to values between 0 and 10, while High category to values between 10.1 and 76.

n: number.

**Supplementary Table S2.** Univariate logistic regression analyses.

**Variables**	**High STAI**	**High STAI-S**	**High STAI-T**	**High GHQ28**	**High GHQ28-A Somatic Symptoms**	**High GHQ28-B Anxiety/insomnia**	**High GHQ28-C Social Dysfunction**	**High GHQ28-D Severe depression**
	(1)	(2)	(3)	(4)	(5)	(6)	(7)	(8)
	OR, 95% CI, *p*-value	OR, 95% CI, *p*-value	OR, 95% CI, *p*-value	OR, 95% CI, *p*-value	OR, 95% CI, *p*-value	OR, 95% CI, *p*-value	OR, 95% CI, *p*-value	OR, 95% CI, *p*-value
Female	2.27 (0.89 - 5.83) p=0.087	1.60 (0.65 - 3.97) p=0.306	**3.54 (1.29 - 9.75) p=0.014**	**3.71 (1.35 - 10.20) p=0.011**	**3.10 (1.13 - 8.52) p=0.029**	**2.75 (1.04 - 7.27) p=0.041**	1.90 (0.74 - 4.87) p=0.182	2.85 (0.97 - 8.31) p=0.056
Age in years	0.96 (0.90 - 1.02) p=0.181	**0.94 (0.88 - 1.00) p=0.045**	0.97 (0.91 - 1.03) p=0.327	1.03 (0.97 - 1.09) p=0.383	0.98 (0.92 - 1.05) p=0.628	0.99 (0.93 - 1.05) p=0.735	**1.08 (1.02 - 1.15) p=0.014**	1.01 (0.95 - 1.08) p=0.703
Married	1.41 (0.66 - 3.01) p=0.377	1.58 (0.74 - 3.39) p=0.237	0.74 (0.35 - 1.57) p=0.427	0.58 (0.27 - 1.24) p=0.161	0.71 (0.33 - 1.53) p=0.385	0.63 (0.30 - 1.36) p=0.240	**0.24 (0.11 - 0.54) p=0.001**	0.50 (0.23 - 1.09) p=0.082
Roommates	1.13 (0.72 - 1.77) p=0.609	**1.62 (1.02 - 2.57) p=0.042**	1.07 (0.68 - 1.69) p=0.761	1.01 (0.65 - 1.59) p=0.953	1.28 (0.81 - 2.03) p=0.298	1.02 (0.65 - 1.60) p=0.937	0.93 (0.59 - 1.46) p=0.752	0.91 (0.57 - 1.46) p=0.695
Education	0.83 (0.51 - 1.35)	0.96 (0.59 - 1.55)	0.77 (0.48 - 1.26)	0.74 (0.45 - 1.20)	0.75 (0.46 - 1.22)	0.68 (0.42 - 1.11)	0.62 (0.37 - 1.03)	0.66 (0.39 - 1.12)
(ISCED Levels)	p=0.457	p=0.868	p=0.304	p=0.222	p=0.243	p=0.127	p=0.065	p=0.122
Income	0.71 (0.44 - 1.15) p=0.161	0.89 (0.56 - 1.43) p=0.640	0.63 (0.39 - 1.02) p=0.063	0.75 (0.47 - 1.21) p=0.242	1.01 (0.63 - 1.62) p=0.953	0.85 (0.53 - 1.36) p=0.492	**0.59 (0.37 - 0.97) p=0.037**	0.67 (0.40 - 1.10) p=0.116
Residency in	1.07 (0.47 - 2.40)	0.81 (0.36 - 1.84)	0.79 (0.35 - 1.80)	0.63 (0.28 - 1.45)	**0.24 (0.09 - 0.61)**	0.46 (0.20 - 1.08)	0.65 (0.28 - 1.49)	0.81 (0.34 - 1.90)
Rural region	p=0.879	p=0.619	p=0.580	p=0.280	**p=0.003**	p=0.076	p=0.307	p=0.622
BMI in kg/m2	1.03 (0.95 - 1.12) p=0.501	1.04 (0.96 - 1.14) p=0.312	1.01 (0.93 - 1.10) p=0.784	1.01 (0.93 - 1.10) p=0.802	1.03 (0.94 - 1.12) p=0.534	0.95 (0.88 - 1.04) p=0.287	0.97 (0.90 - 1.06) p=0.544	1.06 (0.96 - 1.17) p=0.223
BMI Category	1.28 (0.77 - 2.13) p=0.346	1.27 (0.76 - 2.11) p=0.362	1.20 (0.72 - 2.00) p=0.484	1.20 (0.72 - 1.99) p=0.495	1.14 (0.68 - 1.91) p=0.622	0.70 (0.42 - 1.18) p=0.181	1.06 (0.64 - 1.77) p=0.810	1.72 (0.98 - 3.03) p=0.061
Smoker	1.41 (0.57 - 3.50) p=0.456	1.29 (0.52 - 3.20) p=0.580	0.97 (0.39 - 2.40) p=0.942	0.60 (0.24 - 1.52) p=0.281	0.45 (0.17 - 1.20) p=0.109	0.63 (0.25 - 1.59) p=0.325	0.45 (0.17 - 1.20) p=0.109	1.29 (0.51 - 3.26) p=0.584
Previous Smoker	**0.31 (0.10 - 0.91) p=0.034**	0.38 (0.13 - 1.07) p=0.067	**0.23 (0.07 - 0.74) p=0.014**	0.41 (0.14 - 1.17) p=0.094	0.49 (0.17 - 1.38) p=0.176	0.43 (0.15 - 1.22) p=0.111	0.36 (0.12 - 1.08) p=0.070	0.37 (0.11 - 1.19) p=0.096
Years of disease	1.03 (0.99 - 1.07) p=0.111	1.03 (0.99 - 1.07) p=0.109	1.03 (0.99 - 1.07) p=0.134	**1.05 (1.01 - 1.09) p=0.014**	1.04 (1.00 - 1.08) p=0.052	1.02 (0.98 - 1.06) p=0.257	1.03 (1.00 - 1.07) p=0.089	1.04 (1.00 - 1.08) p=0.051
Years of disease above median	**2.15 (1.01 - 4.59) p=0.047**	**2.55 (1.19 - 5.46) p=0.017**	1.72 (0.81 - 3.63) p=0.159	**2.50 (1.17 - 5.36) p=0.019**	1.81 (0.85 - 3.84) p=0.125	1.72 (0.81 - 3.63) p=0.159	1.56 (0.74 - 3.31) p=0.246	**2.76 (1.26 - 6.08) p=0.011**
Hypertension	0.54 (0.25 - 1.14) p=0.106	0.71 (0.33 - 1.49) p=0.360	0.78 (0.37 - 1.64) p=0.509	1.27 (0.60 - 2.68) p=0.526	1.14 (0.54 - 2.42) p=0.725	0.90 (0.43 - 1.89) p=0.776	1.14 (0.54 - 2.42) p=0.725	1.24 (0.57 - 2.70) p=0.580
Diabetes	0.89 (0.34 - 2.36) p=0.816	0.82 (0.31 - 2.17) p=0.686	1.94 (0.72 - 5.21) p=0.189	1.45 (0.55 - 3.84) p=0.455	1.06 (0.40 - 2.80) p=0.910	0.73 (0.27 - 1.95) p=0.524	1.35 (0.51 - 3.57) p=0.545	0.69 (0.24 - 1.97) p=0.488
Thyroid disease	0.84 (0.34 - 2.06) p=0.704	1.42 (0.58 - 3.49) p=0.439	0.58 (0.23 - 1.44) p=0.239	0.84 (0.34 - 2.06) p=0.704	1.01 (0.41 - 2.47) p=0.987	0.71 (0.29 - 1.76) p=0.464	0.66 (0.26 - 1.65) p=0.374	0.95 (0.38 - 2.41) p=0.922
Cancer	3.47 (0.35 - 34.76) p=0.290	1.04 (0.14 - 7.69) p=0.972	3.60 (0.36 - 36.06) p=0.276	-	4.02 (0.40 - 40.29) p=0.237	-	4.02 (0.40 - 40.29) p=0.237	-
Lipidaemia	0.82 (0.39 - 1.76) p=0.615	0.73 (0.34 - 1.56) p=0.422	1.01 (0.47 - 2.16) p=0.976	1.11 (0.52 - 2.35) p=0.794	1.21 (0.56 - 2.58) p=0.627	0.87 (0.41 - 1.86) p=0.725	**2.20 (1.02 - 4.75) p=0.045**	0.91 (0.42 - 2.00) p=0.819
Osteoporosis	0.70 (0.29 - 1.69) p=0.433	0.64 (0.27 - 1.55) p=0.323	0.90 (0.38 - 2.15) p=0.808	0.70 (0.29 - 1.69) p=0.433	**0.36 (0.14 - 0.94) p=0.036**	0.60 (0.25 - 1.47) p=0.264	2.27 (0.94 - 5.49) p=0.070	1.01 (0.41 - 2.48) p=0.981
Inflamm Bowel Disease	2.27 (0.20 - 26.03) p=0.510	2.11 (0.18 - 24.22) p=0.548	-	2.27 (0.20 - 26.03) p=0.510	0.63 (0.06 - 7.26) p=0.713	0.57 (0.05 - 6.51) p=0.649	0.63 (0.06 - 7.26) p=0.713	3.55 (0.31 - 40.82) p=0.309
Fibromyalgia	1.94 (0.69 - 5.45) p=0.210	2.36 (0.82 - 6.85) p=0.113	1.54 (0.56 - 4.26) p=0.405	1.13 (0.41 - 3.12) p=0.808	**3.05 (1.05 - 8.87) p=0.040**	2.02 (0.72 - 5.68) p=0.183	1.75 (0.63 - 4.85) p=0.282	0.83 (0.29 - 2.43) p=0.738
COPD	1.64 (0.49 - 5.53) p=0.427	1.51 (0.45 - 5.11) p=0.504	**3.95 (1.00 - 15.56) p=0.049**	3.80 (0.97 - 14.95) p=0.056	1.92 (0.57 - 6.50) p=0.294	1.70 (0.50 - 5.76) p=0.391	1.32 (0.40 - 4.39) p=0.653	1.83 (0.55 - 6.13) p=0.325
No. of Comorbidities	0.98 (0.75 - 1.27) p=0.858	1.00 (0.77 - 1.29) p=0.986	1.10 (0.86 - 1.43) p=0.446	1.12 (0.87 - 1.44) p=0.393	1.13 (0.87 - 1.45) p=0.361	1.02 (0.79 - 1.31) p=0.905	1.21 (0.93 - 1.57) p=0.155	1.13 (0.86 - 1.48) p=0.373
No. of Comorbidities above median	0.90 (0.43 - 1.90) p=0.780	1.06 (0.50 - 2.24) p=0.874	1.28 (0.60 - 2.70) p=0.524	1.20 (0.57 - 2.54) p=0.633	1.15 (0.54 - 2.44) p=0.718	0.96 (0.45 - 2.02) p=0.905	1.54 (0.72 - 3.27) p=0.263	1.67 (0.77 - 3.61) p=0.196
ESR	**1.04 (1.01 - 1.07) p=0.015**	**1.03 (1.00 - 1.06) p=0.024**	**1.03 (1.01 - 1.06) p=0.019**	1.03 (1.00 - 1.06) p=0.056	1.01 (0.98 - 1.03) p=0.609	1.01 (0.98 - 1.03) p=0.696	1.01 (0.98 - 1.04) p=0.453	1.00 (0.97 - 1.02) p=0.772
CRP	1.15 (0.60 - 2.20) p=0.680	1.08 (0.58 - 2.02) p=0.797	1.27 (0.60 - 2.67) p=0.530	1.22 (0.60 - 2.47) p=0.577	0.94 (0.55 - 1.61) p=0.830	1.04 (0.58 - 1.85) p=0.899	0.93 (0.55 - 1.59) p=0.798	0.90 (0.55 - 1.46) p=0.661
RF	1.29 (0.61 - 2.73) p=0.500	1.14 (0.54 - 2.41) p=0.726	0.58 (0.27 - 1.23) p=0.155	0.63 (0.30 - 1.33) p=0.227	0.52 (0.24 - 1.11) p=0.090	0.50 (0.23 - 1.07) p=0.073	0.93 (0.44 - 1.97) p=0.852	**0.38 (0.17 - 0.86) p=0.020**
CCP	1.37 (0.63 - 3.01) p=0.429	1.45 (0.66 - 3.18) p=0.357	0.65 (0.29 - 1.44) p=0.292	0.62 (0.28 - 1.37) p=0.236	0.46 (0.20 - 1.05) p=0.065	0.90 (0.41 - 1.97) p=0.792	0.90 (0.41 - 1.98) p=0.790	0.71 (0.31 - 1.62) p=0.413
HAQ-DI	**2.33 (1.28 - 4.24) p=0.006**	**1.78 (1.02 - 3.09) p=0.042**	**3.44 (1.78 - 6.66) p<0.001**	**4.57 (2.08 - 10.01) p<0.001**	**8.15 (3.05 - 21.80) p<0.001**	**2.45 (1.33 - 4.49) p=0.004**	**3.65 (1.75 - 7.65) p=0.001**	**1.97 (1.13 - 3.41) p=0.016**
HAQ-DI scale (2 Categories)	1.76 (0.79 - 3.96) p=0.168	1.14 (0.51 - 2.54) p=0.744	**2.62 (1.15 - 5.98) p=0.022**	**4.31 (1.81 - 10.27) p=0.001**	**10.36 (3.94 - 27.27) p<0.001**	**2.62 (1.15 - 5.98) p=0.022**	**3.69 (1.59 - 8.57) p=0.002**	1.44 (0.64 - 3.28) p=0.379
CDAI	**1.06 (1.00 - 1.13) p=0.037**	**1.06 (1.00 - 1.12) p=0.050**	**1.06 (1.00 - 1.11) p=0.049**	**1.08 (1.02 - 1.15) p=0.010**	**1.12 (1.04 - 1.21) p=0.004**	1.05 (1.00 - 1.11) p=0.056	**1.07 (1.02 - 1.14) p=0.012**	1.00 (0.96 - 1.05) p=0.972
CDAI scale (2 Categories)	**2.96 (1.35 - 6.49) p=0.007**	**2.63 (1.20 - 5.74) p=0.016**	**2.69 (1.23 - 5.86) p=0.013**	**3.48 (1.57 - 7.71) p=0.002**	**3.82 (1.72 - 8.48) p=0.001**	**2.69 (1.23 - 5.86) p=0.013**	**3.25 (1.47 - 7.14) p=0.003**	1.55 (0.71 - 3.39) p=0.269
**Treatments**								
Hydroxycloroquine	1.27 (0.52 - 3.10) p=0.602	1.16 (0.48 - 2.83) p=0.746	1.08 (0.44 - 2.64) p=0.865	1.03 (0.42 - 2.52) p=0.943	1.01 (0.41 - 2.47) p=0.987	1.08 (0.44 - 2.64) p=0.865	0.82 (0.33 - 2.02) p=0.661	0.76 (0.30 - 1.96) p=0.572
Methotrexate	1.44 (0.69 - 3.03) p=0.335	1.24 (0.59 - 2.60) p=0.569	1.35 (0.64 - 2.83) p=0.431	0.94 (0.45 - 1.97) p=0.875	1.10 (0.52 - 2.32) p=0.797	0.76 (0.36 - 1.61) p=0.478	1.97 (0.92 - 4.20) p=0.079	0.98 (0.46 - 2.12) p=0.967
Leflunomide	0.73 (0.12 - 4.58) p=0.738	1.58 (0.25 - 9.94) p=0.623	0.76 (0.12 - 4.76) p=0.768	1.71 (0.27 - 10.70) p=0.569	0.85 (0.13 - 5.32) p=0.860	0.27 (0.03 - 2.56) p=0.256	0.31 (0.03 - 2.86) p=0.299	0.41 (0.04 - 3.88) p=0.440
anti-TNF	**2.60 (1.04 - 6.51) p=0.041**	**4.81 (1.75 - 13.23) p=0.002**	1.47 (0.61 - 3.54) p=0.396	2.11 (0.86 - 5.18) p=0.105	2.06 (0.85 - 5.04) p=0.111	1.47 (0.61 - 3.54) p=0.396	**3.15 (1.25 - 7.90) p=0.015**	2.50 (1.02 - 6.12) p=0.045
anti-IL6	0.22 (0.04 - 1.07) p=0.060	**0.20 (0.04 - 0.99) p=0.048**	0.40 (0.10 - 1.59) p=0.193	0.22 (0.04 - 1.07) p=0.060	0.25 (0.05 - 1.25) p=0.091	0.40 (0.10 - 1.59) p=0.193	0.45 (0.11 - 1.79) p=0.255	0.15 (0.02 - 1.24) p=0.078
Jak-inhibitors	0.73 (0.12 - 4.58) p=0.738	0.25 (0.03 - 2.29) p=0.218	0.76 (0.12 - 4.76) p=0.768	0.73 (0.12 - 4.58) p=0.738	0.85 (0.13 - 5.32) p=0.860	1.77 (0.28 - 11.11) p=0.542	1.98 (0.32 - 12.42) p=0.467	0.41 (0.04 - 3.88) p=0.440
anti-CD20	4.82 (0.52 - 44.96) p=0.168	1.62 (0.26 - 10.14) p=0.609	5.00 (0.54 - 46.68) p=0.158	4.82 (0.52 - 44.96) p=0.168	5.60 (0.60 - 52.31) p=0.131	-	5.39 (0.58 - 50.35) p=0.139	2.76 (0.44 - 17.41) p=0.279
glucocorticoids	1.39 (0.66 - 2.94) p=0.384	0.92 (0.44 - 1.94) p=0.833	1.29 (0.61 - 2.71) p=0.509	1.61 (0.76 - 3.40) p=0.213	1.35 (0.64 - 2.85) p=0.434	1.29 (0.61 - 2.71) p=0.509	2.09 (0.98 - 4.47) p=0.056	1.09 (0.51 - 2.36) p=0.822
No. of Treatments	**1.70 (1.01 - 2.83) p=0.044**	1.36 (0.83 - 2.23) p=0.221	1.45 (0.89 - 2.38) p=0.139	1.48 (0.89 - 2.48) p=0.132	1.36 (0.82 - 2.26) p=0.233	1.13 (0.69 - 1.85) p=0.637	**2.71 (1.63 - 4.51) p<0.001**	1.08 (0.66 - 1.77) p=0.764
No. of Treatments above median	2.80 (0.80 - 9.74) p=0.106	2.59 (0.74 - 8.99) p=0.135	2.91 (0.84 - 10.15) p=0.093	**7.42 (1.55 - 35.46) p=0.012**	**5.08 (1.31 - 19.73) p=0.019**	1.99 (0.61 - 6.54) p=0.256	**8.74 (1.83 - 41.85) p=0.007**	1.08 (0.33 - 3.57) p=0.898

High levels for each outcome were considered values above 50% percentile (median value). All Previous Treatments had insignificant univariate associations with the outcomes.

BMI: body mass index; kg: kilogram; m: meter; COPD: chronic obstructive pulmonary disease; ESR: Erythrocyte Sedimentation Rate; CRP: C-reactive protein; RF: Rheumatoid Factor; CCP: Cyclic Citrullinated Peptide; HAQ-DI: Health Assessment Questionnaire Disability Index; CDAI: Clinical Disease Activity Index.
